# Rosette cataract thirty years after trauma

**DOI:** 10.11604/pamj.2024.47.140.42928

**Published:** 2024-03-26

**Authors:** Amine Zahaf, Nada Bouallegui

**Affiliations:** 1 Department of Ophthalmology, Internal Security Forces Hospital, 29 Rue Tahar Ben Achour, La Marsa, Tunis, Tunisia,; 2 Faculty of Medicine of Tunis, University of Tunis El Manar, 15 Rue Djebel Lakhdhar, Tunis, Tunisia

**Keywords:** Cataract, ocular trauma, ocular injuries

## Image in medicine

A 48-year-old healthy woman presented with complaints of diminution of vision in the right eye for the last 8 months. She reported having undergone trauma to her right eye at the age of 18. Visual acuity was 20/40 right eye. The slit lamp biomicroscopy of the right eye showed a central endonucleus cataract with white axial opacities organized in a distinctive Rosette pattern, forming six distinct quadrangular 'petals´ (yellow arrow) and a transparent outer epinuclear shell (blue arrow). Notably, the absence of zonular rupture differentiates it from a subluxated cataract. Cataracts with a Rosette or stellate-shaped appearance are typically seen in blunt or ocular injuries. Prior to surgery, patients must be checked for associated lesions, especially zonular dehiscence.

**Figure 1:**
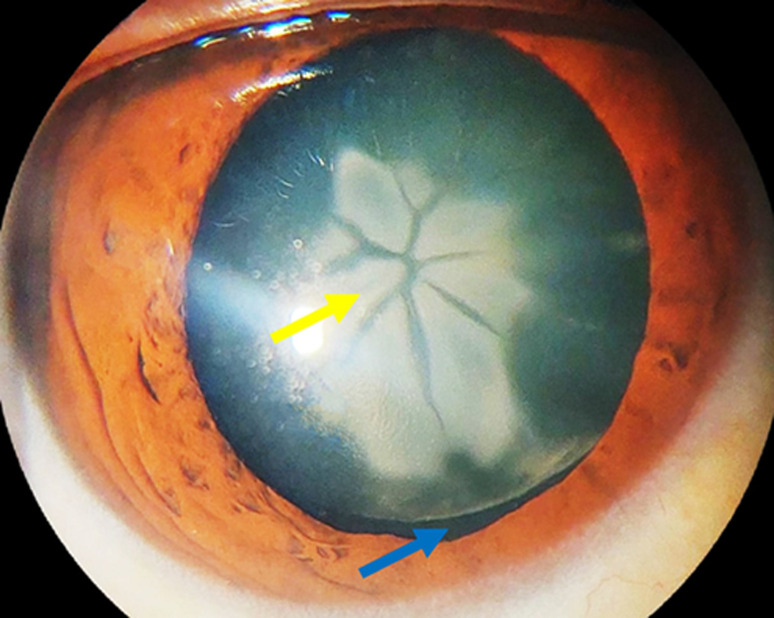
central endonucleus cataract with white axial opacities organized in a distinctive Rosette pattern, forming six distinct quadrangular ´petals´ (yellow arrow) and transparent outer epinuclear shells without zonular dehiscence (blue arrow)

